# Supplementary material for: MetaRibo-Seq measures translation in microbiomes
Source: Nat Commun. 2020 Jun 29;11:3268. doi: 10.1038/s41467-020-17081-z (PMC7324362; doi:10.1038/s41467-020-17081-z)
Supplement: Supplementary file 10 — Supplementary Data 7 [file 41467_2020_17081_MOESM10_ESM.zip › File2/Confidence_High_Taxonomy/368825_out.krona.html]

Javascript must be enabled to view this page.

members
magnitude
magnitudeUnassigned
count
unassigned
taxon
rank

368825\_out

40

superkingdom
2
40

phylum
40
976

class
200643
40

40
171549
order

3
815
family

genus
816
3

371601
1

SRS064973\_contig\_number\_410
species

28111
1
species

SRS018575\_contig\_number\_7335


SRS893252\_contig\_number\_17395
species
1
2292944

2005525
36
family

375288
36

SRS014287\_contig\_number\_19882SRS015663\_contig\_number\_35633SRS045244\_contig\_number\_7394SRS056519\_contig\_number\_15487SRS143085\_contig\_number\_contig-100\_392.33493SRS148970\_contig\_number\_352
6
genus

species

SRS043667\_contig\_number\_1005SRS098717\_contig\_number\_7188
2
46503

22
823
species

SRS014459\_contig\_number\_11340SRS015065\_contig\_number\_contig-100\_229.221655SRS015217\_contig\_number\_31586SRS015579\_contig\_number\_25437SRS015890\_contig\_number\_5820SRS018984\_contig\_number\_1771SRS019397\_contig\_number\_8493SRS019968\_contig\_number\_2557SRS046369\_contig\_number\_477SRS049995\_contig\_number\_29639SRS051610\_contig\_number\_10995SRS051882\_contig\_number\_10589SRS054590\_contig\_number\_230SRS062701\_contig\_number\_6088SRS075878\_contig\_number\_7230SRS077454\_contig\_number\_19357SRS1041091\_contig\_number\_16263SRS1041142\_contig\_number\_2491SRS1055038\_contig\_number\_6007SRS142712\_contig\_number\_2672SRS148511\_contig\_number\_16627SRS893382\_contig\_number\_6721

2293123
6

SRS015431\_contig\_number\_35473SRS019068\_contig\_number\_53025SRS020622\_contig\_number\_4381SRS051031\_contig\_number\_24250SRS063370\_contig\_number\_13010SRS1041090\_contig\_number\_5079
species

1853231
1
family

genus
283168
1

species

SRS058770\_contig\_number\_23363
28118
1
